# Real-Time Prediction of Irreversible Lesion Size During Pulsed Field Ablation: Prospective Validation of a Novel Ablation Index Based on Contact Force and Number of Applications in a Swine Beating Heart Model

**DOI:** 10.1161/CIRCEP.125.013911

**Published:** 2025-12-02

**Authors:** Hiroshi Nakagawa, Salman Farshchi-Heydari, Masafumi Sugawara, Atsushi Ikeda, Jennifer Maffre, Tushar Sharma, Philip Lam, Assaf Govari, Christopher T. Beeckler, Andres Altmann, Warren M. Jackman, Michael R. Franz, Taylor Spangler, Ayman A. Hussein, Shady Nakhla, Pasquale Santangeli, Walid I. Saliba, Oussama M. Wazni

**Affiliations:** 1 Department of Cardiovascular Medicine, Cleveland Clinic, OH (H.N., M.S., A.A.H., S.N., P.S., W.I.S., O.M.W.).; 2 Johnson & Johnson MedTech, Irwindale, CA (S.F.-H., J.M., T. Sharma, P.L., C.T.B.).; 3 Department of Cardiology, Nihon University, Japan (A.I.).; 4 Johnson & Johnson MedTech, Yokneam, Israel (A.G., A.A.).; 5 Heart Rhythm Institute, University of Oklahoma (W.M.J.).; 6 Georgetown University & Veteran Affairs Medical Centers (M.R.F.).; 7 Bayside Preclinical Services, Davis, CA (T. Spangler).

**Keywords:** atrial fibrillation, catheter ablation, irreversible electroporation, pulsed-field ablation, ventricular tachycardia

## Abstract

**BACKGROUND:**

In a previous study, on pulsed-field ablation (PFA) in the swine ventricle, we found that lesion depth was described (±1 mm accuracy) by a logarithmic function of contact force (CF) and the number of PFA pulses (PF-ablation index). This study was designed to validate prospectively whether the novel PF-ablation index would allow PFA lesions to be created at depths of 3.5, 4.5, 5.5, and 6.5 mm with high prediction accuracy in a swine-beating heart model.

**METHODS:**

A 7.5F catheter with a 3.5 mm ablation electrode and CF sensor (ThermoCool SmartTouch SF-Dual Energy) was connected to a PFA system (TRUPULSE 2). In 6 closed-chest swine, a biphasic PFA pulse was delivered between the ablation electrode and a skin patch at 123 separate ventricular sites at 5 different levels of CF (1) low (average CF: 4–15 g; median, 12 g; n=25), (2) moderate (16–30 g; median, 23 g; n=41); 3) high (31–45 g; median, 36 g; n=27), (4) very high (46–68 g; median, 52 g; n=18); or (5) no electrode contact, 1 to 2 mm from the endocardium (n=12). PFA application was terminated when the PF-ablation index reached a predicted lesion depth of 3.5 mm (27 sites), 4.5 mm (25 sites), 5.5 mm (29 sites), and 6.5 mm (30 sites). Swine were euthanized 2 hours after ablation. Lesion size was measured using triphenyl tetrazolium chloride staining.

**RESULTS:**

Predicted lesion depth by the PF-ablation index correlated well with actual lesion depthwith ±1.0 mm accuracy in 97/106 (92%) lesions and ±1.5 mm accuracy in all 106 lesions. There were no or poor relationships between intracardiac electrogram attenuation, impedance decrease, electrode temperature, and lesion size. No detectable lesions were created without electrode contact.

**CONCLUSIONS:**

A novel PF-ablation index incorporating CF and the number of PFA pulses provides high accuracy in predicting lesion depth in real-time. Intracardiac electrogram attenuation, impedance decrease, and electrode temperature are poor predictors of PFA lesion size.

**WHAT IS KNOWN?**

- In contrast to radiofrequency ablation, it has been suggested that pulsed field ablation (PFA) does not require electrode-tissue contact to achieve effective ablation lesions.
- However, recent studies in a swine beating heart model have shown: (1) electrode-tissue contact is required for irreversible PFA lesion formation and (2) lesion depth increases significantly with increasing contact force and the number of PFA applications.

**WHAT THE STUDY ADDS**

- In this prospective validation study using the biphasic monopolar focal PFA system in a closed chest beating heart swine model, a novel PF Ablation Index (incorporating contact force and the number of PFA pulses) provided real-time prediction of PFA lesion depth with high accuracy, with ±1.0 mm accuracy in 97/106 (92%) lesions and ±1.5 mm accuracy in all 106 lesions.
- No detectable lesion formation without electrode-tissue contact, indicating the requirement of electrode-tissue contact for effective PFA lesion formation.
- There was no or only a poor relationship between changes in the intracardiac electrogram after PFA (attenuation of bipolar and unipolar electrogram amplitude and mean negative *dV/dt,* or ST-segment–elevation) and lesion size, and no or only a poor correlation between impedance decrease or maximum electrode temperature and lesion size.

The size of ablation lesions created by radiofrequency applications increases with increasing radiofrequency power, application time, and contact force (CF).^1–4^

A logarithmic formula based on a combination of CF, radiofrequency power, and application time (force-power-time index, ablation index) has been developed to predict lesion size during radiofrequency ablation. The ablation index allowed the real-time prediction of radiofrequency lesion depth in the 3 to 9 mm range with high accuracy in beating canine ventricles. Several clinical studies using the ablation index have demonstrated its usefulness in controlling lesion size, including the high incidence of pulmonary vein (PV) isolation with the first PV encirclement.^5,6^

Pulsed-field ablation (PFA) is a modality that produces primarily nonthermal lesions by delivering high electric current with very short pulse applications, leading to cell membrane permeabilization, apoptosis, and cell death.^7–12^ In contrast to radiofrequency, it has been suggested that PFA may not require electrode-tissue contact to achieve effective ablation lesions because intracardiac electrograms are often eliminated by PFA without electrode-tissue contact.^13,14^ However, recent studies in a swine beating heart model have shown: (1) electrode-tissue contact is required for irreversible PFA lesion formation; (2) lesion depth increases significantly with increasing CF and the number of PFA applications; and (3) a logarithmic formula combined with CF and the number of PFA pulses correlates well with lesion depth.^15–17^

The purpose of the present study was to use a swine beating heart model to (1) prospectively validate the accuracy of the real-time prediction of PFA lesion depth using a novel logarithmic formula (PF ablation index); (2) determine the relationships between intracardiac electrogram attenuation recorded from the ablation electrode and lesion size; and (3) determine the relationships between impedance decrease, electrode temperature, and lesion size.

## Methods

The data supporting this study’s findings are available from the corresponding author on reasonable request.

### Pulsed-Field Ablation Catheter and System

This study utilized a 7.5Fr, quadripolar catheter with a 3.5 mm saline irrigated tip ablation electrode containing a magnetic CF sensor and an internal thermocouple (ThermoCool SmartTouch SF-Dual Energy, Johnson &Johnson MedTech Inc, Irwindale, CA). A PFA generator system (TRUPULSE 2, Johnson &Johnson MedTech Inc) was employed to deliver high voltage, biphasic, monopolar, microsecond sinusoidal waveforms between the ablation electrode and a skin patch during saline irrigation of the ablation electrode at 4 mL/min.^17^

### Novel Pulsed Field Ablation Index Formula

In the previous study, PFA was performed in a swine ventricular model with combinations of CF (4–65 g) and PFA dose (12, 18, and 24 burst pulses).^17^ Lesion depth increased significantly with increasing CF and the number of PFA burst pulses. Multivariable regression analysis, retrospectively, assessed the relationship between lesion depth and the combination of average CF and the number of burst pulses to produce a logarithmic formula. Lesion depth calculated by the logarithmic formula correlated well with actual lesion depth with high accuracy, *R*=0.809, *R*^2^=0.655, *P*<0.0001, Y=0.975×X+0.216, ±1.0 mm accuracy in 128/163 (79%) lesions and ±1.5 mm accuracy in 153/163 (94%) lesions.^17^

After additional data from other preclinical experiments were also taken into account, the following novel logarithmic formula was developed.


PFAblationIndex=A*[B(n)*log(n)+C(n)]

*A*, *B*, and *C* are constant, and *n* is a combination of the average CF and the number of PFA pulses. The index value is displayed as the predicted lesion depth (mm) multiplied by 100. For example, if the predicted lesion depth is 5.5 mm, the index value is displayed as 550.

The PF ablation index formula was integrated into the 3-dimensional mapping system (CARTO3, Johnson &Johnson MedTech), allowing the predicted lesion depth to be displayed in real-time during PFA applications.

### Prospective Validation of a Novel PF Ablation Index Based on Contact Force and Number of Applications for Prediction of Lesion Depth

#### Experimental Preparation

The experimental protocol was approved by the Committee on the Use and Care of Animals at the Global Medical Laboratory, Pomona, CA. Six Yorkshire swine (median body weight of 87.8 kg) were anesthetized with isoflurane and mechanically ventilated. No neuromuscular paralytic agents were administered. A 6F decapolar catheter was inserted into the right jugular vein and advanced into the coronary sinus. An 8.5F ultrasound catheter (AcuNav, Acuson, Mountain View, CA) was inserted into the left femoral vein and positioned in the right atrium to be used for intracardiac echocardiography (ICE). Heparin (10 000–12 000 IU) was administered intravenously with additional doses to maintain the activated clotting time ≥300 seconds. Transeptal puncture was performed under ICE and fluoroscopic guidance. A deflectable transeptal sheath (Vizigo, Johnson &Johnson MedTech) was advanced into the left atrium and left ventricle (LV). An anatomic shell of the LV chamber was created using a multi-electrode mapping catheter (Octaray, Johnson &Johnson MedTech) and the CARTO3 mapping system. The mapping catheter was switched to the PF ablation catheter (ThermoCool SmartTouch SF-Dual Energy). The ablation catheter was initially positioned centrally in the left atrium without electrode-tissue contact (confirmed by ICE) to calibrate the CF-sensor to 0 g (baseline noncontact value). The ablation catheter was then advanced into the LV for ablation. The decapolar catheter in the coronary sinus was repositioned into the right ventricular (RV) apex for pacing during PFA. After LV ablation was completed, an anatomic shell of the RV was created with the Octaray catheter, and PFA was then performed in the RV.

#### Ablation Protocol

**Figure 1.**
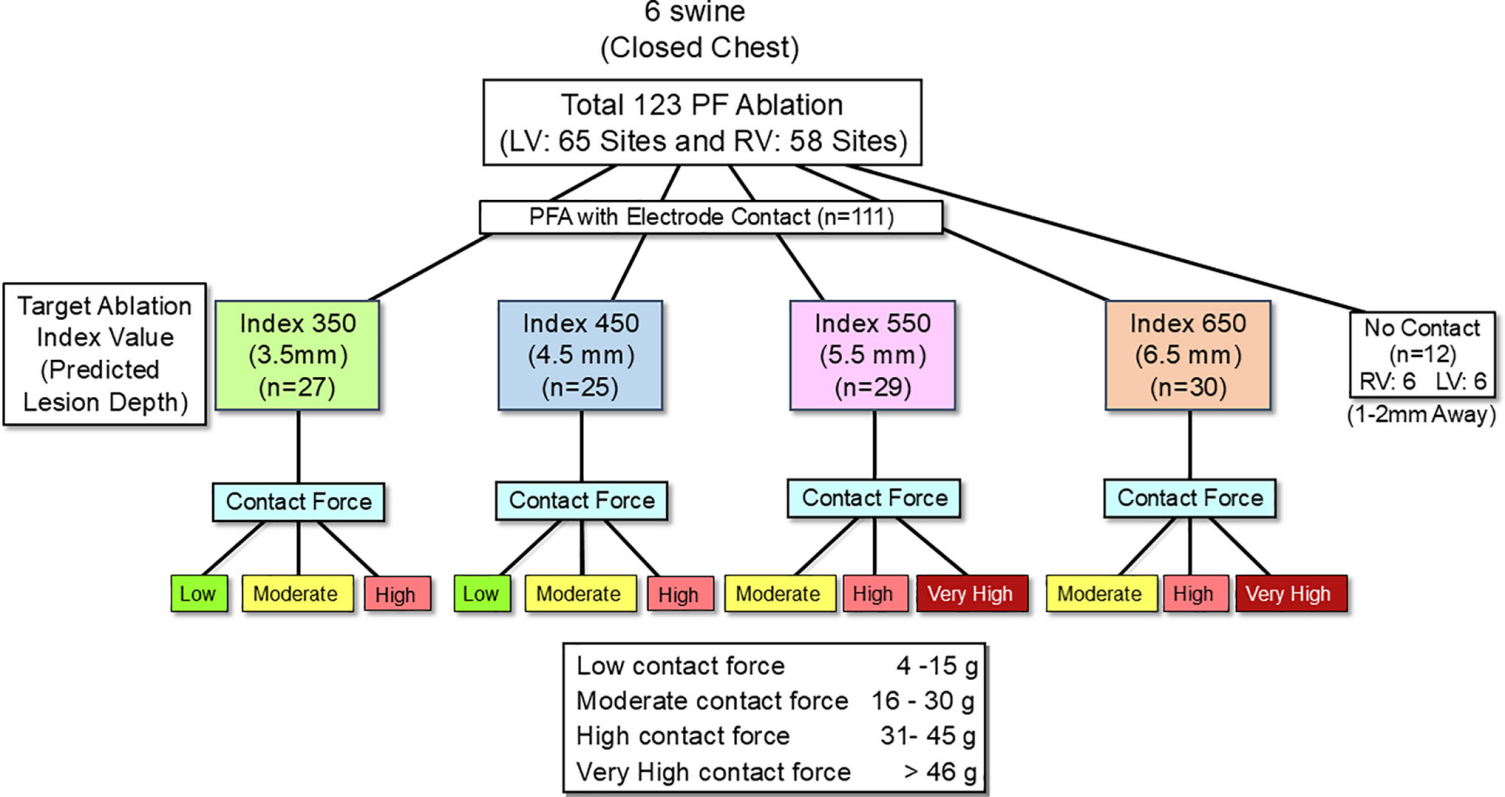
**Protocol for the prospective validation study of a novel pulsed field (PF) ablation (PFA) index to predict lesion depth.** In a total of 6 swine, biphasic monopolar focal PFA was performed at 123 separate ventricular sites in the left ventricle (LV; n=65) and right ventricle (RV; n=58) with target PF ablation index values of 350 (n=27), 450 (n=25), 550 (n=29) and 650 (n=30) with 5 different levels of contact force (CF): (1) low (n=25, average CF, 6–14 g; median, 12 g); (2) moderate (n=43; 16–30 g; median, 23 g); (3) high (n=28; 31–45 g; median, 36 g); (4) very high (n=15; 48–68 g; median, 52 g); and (5) no electrode contact (n=12, ≈1–2 mm away from the endocardium). See the text for details.

**Figure 2.**
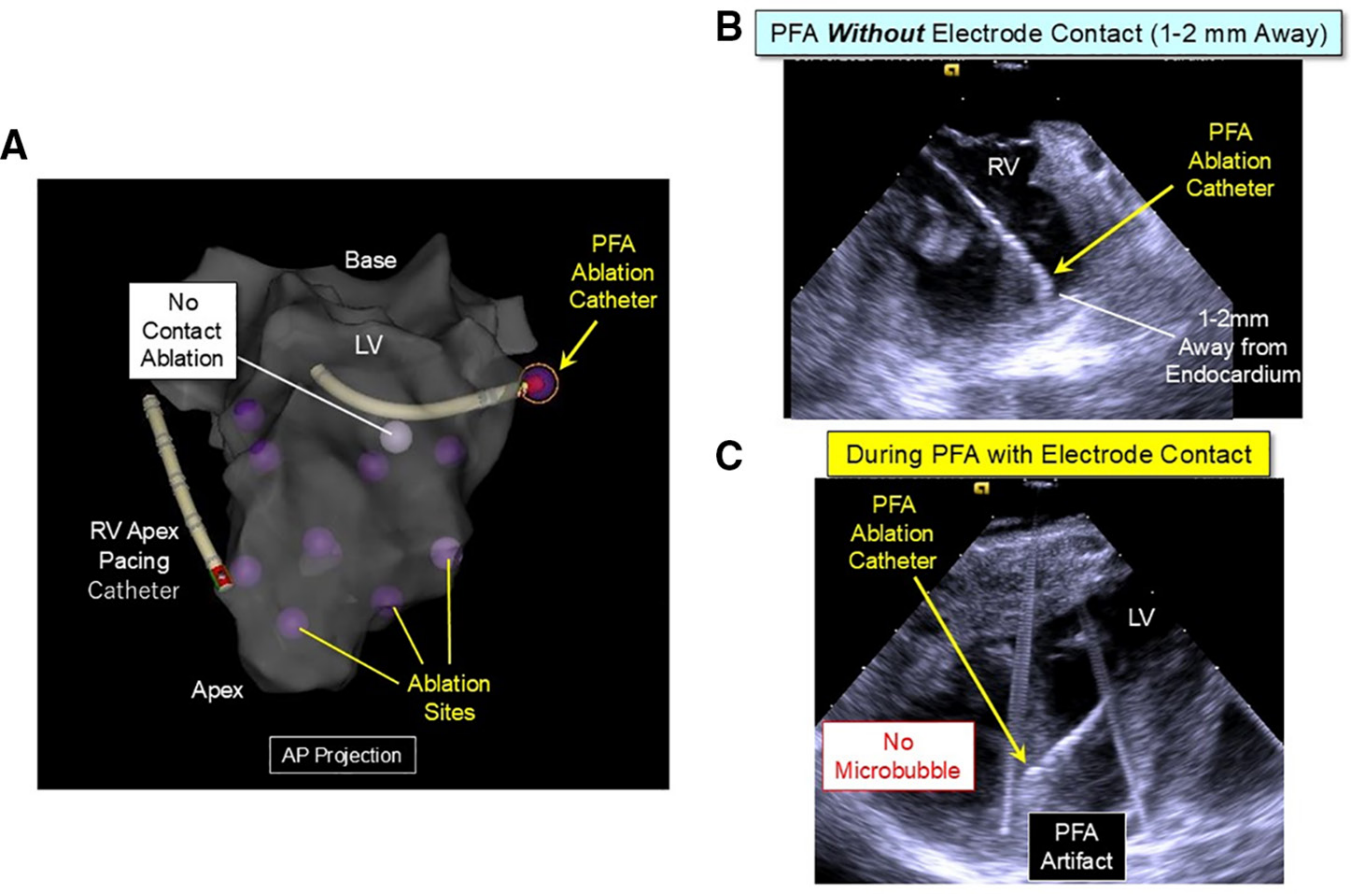
**Three-dimensional electro-anatomic map and intracardiac echocardiography of the ventricle demonstrating pulsed field ablation (PFA) sites with and without electrode contact. A**, The anatomic shell of the left ventricle (LV) is shown in the anterior-posterior (AP) projection. PFA is performed at separate sites with electrode contact (purple tags) and without contact (white tag) during right ventricle (RV) apex pacing. **B**, Intracardiac echocardiography showing a PFA catheter. PFA is performed without electrode-myocardium contact, ≈1 to 2 mm away from the endocardium. **C**, No microbubbles are detected during PFA applications in the LV with electrode contact. White lines indicate the artifact produced by PFA applications.

During RV pacing at a cycle length of 400 ms, biphasic monopolar PFA pulses were delivered in the LV and RV between the ablation electrode and a skin patch (positioned on the posterior chest) to a total of 123 ventricular sites in the 6 swine (Figure 1). Each PFA application consisted of multiple PFA burst pulses without ECG gating. Each PFA burst pulse contains a train of multiple very brief pulses, delivered within the 129-ms window with an average energy of 10.65 Joules.^17^ The maximum number of PFA burst pulses delivered for any application was limited to 24 for this PFA system. In each swine, PFA was performed using 5 different levels of CF: (1) low CF (average 4–15 g); (2) moderate CF (average 16–30 g); (3) high CF (average 31–45 g); (4) very high CF (average >46 g); or (5) no electrode-myocardium contact, with the ablation electrode positioned ≈1 to 2 mm away from the endocardium, confirmed by ICE and CF monitoring (Figures 1 and 2). PFA applications were terminated when the PF Ablation Index reached targeted values of 350 (predicting lesion depth of 3.5 mm), 450 (predicting lesion depth of 4.5 mm), 550 (predicting lesion depth of 5.5 mm), and 650 (predicting lesion depth of 6.5 mm), respectively (Figure 1). The presence or absence of microbubbles during PFA delivery was monitored by ICE (Figure 2, right panel). The ablation locations were stored on the CARTO3 mapping system and were sufficiently separated to be accurately identified during lesion assessment (Figure 2, left panel).

The impedance (measured between the ablation electrode and the skin patch) and electrode temperature were recorded during PFA applications.

#### Measurements of Intracardiac Electrogram Parameters Recorded From the PF Ablation Catheter

**Figure 3.**
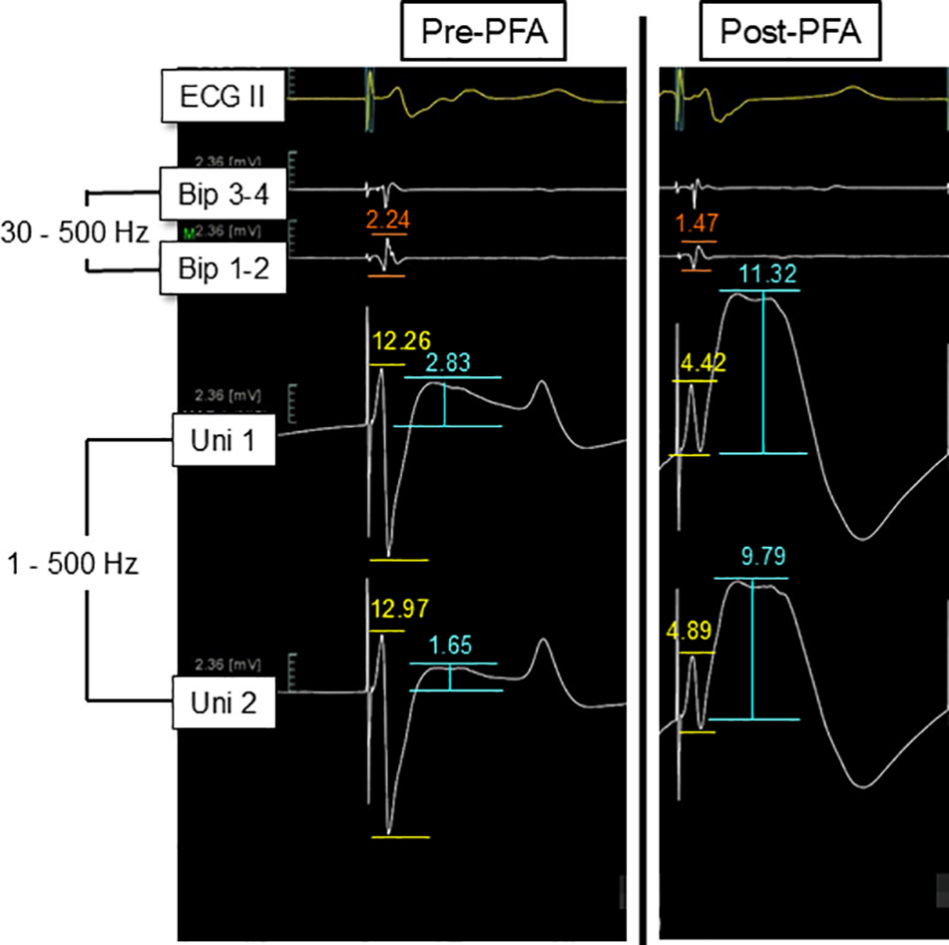
**Measurements of intracardiac electrograms before and after pulsed field ablation (PFA).** During right ventricle (RV) pacing, distal bipolar electrograms (Bip 1–2, filtered at 30–500 Hz) and unipolar electrograms (filtered at 1–500 Hz) are recorded between the distal ablation electrode and the Wilson central terminal (WCT, Uni 1) and between the second electrode and the WCT (Uni 2). Intracardiac electrogram measurements are obtained at each ablation site before and after PFA application: (1) bipolar ventricular potential amplitude (peak-to-peak of the RS segment, orange lines); (2) unipolar ventricular potential amplitude (peak-to-peak of the RS segment) from the distal and second electrodes (yellow lines); (3) mean negative d*V*/d*t* (slope, amplitude/duration of the downstroke) from the distal unipolar electrogram; and (4) unipolar ST-segment amplitude (injury current) from the distal and second electrodes (blues lines). All intracardiac electrograms are shown using the same gain. See the text for details.

Bipolar electrograms were recorded between the distal ablation electrode and the second electrode, filtered at 30 to 500 Hz. Unipolar electrograms were recorded between the distal ablation electrode and the Wilson central terminal, and between the second electrode and the Wilson central terminal, filtered at 1 to 500 Hz (Figure 3). The following intracardiac electrogram measurements were obtained at each ablation site before and after PFA application (1) bipolar ventricular potential amplitude (peak-to-peak of the RS segment); (2) unipolar ventricular potential amplitude (peak-to-peak of the RS segment) from the distal and second electrodes; (3) distal unipolar potential mean negative *dV/dt* (amplitude/duration of the downstroke); and (4) unipolar ST-segment amplitude (injury current) from the distal and second electrodes (Figure 3).

#### Measurements of Lesion Size and Histological Examination of PFA Lesions

**Figure 4.**
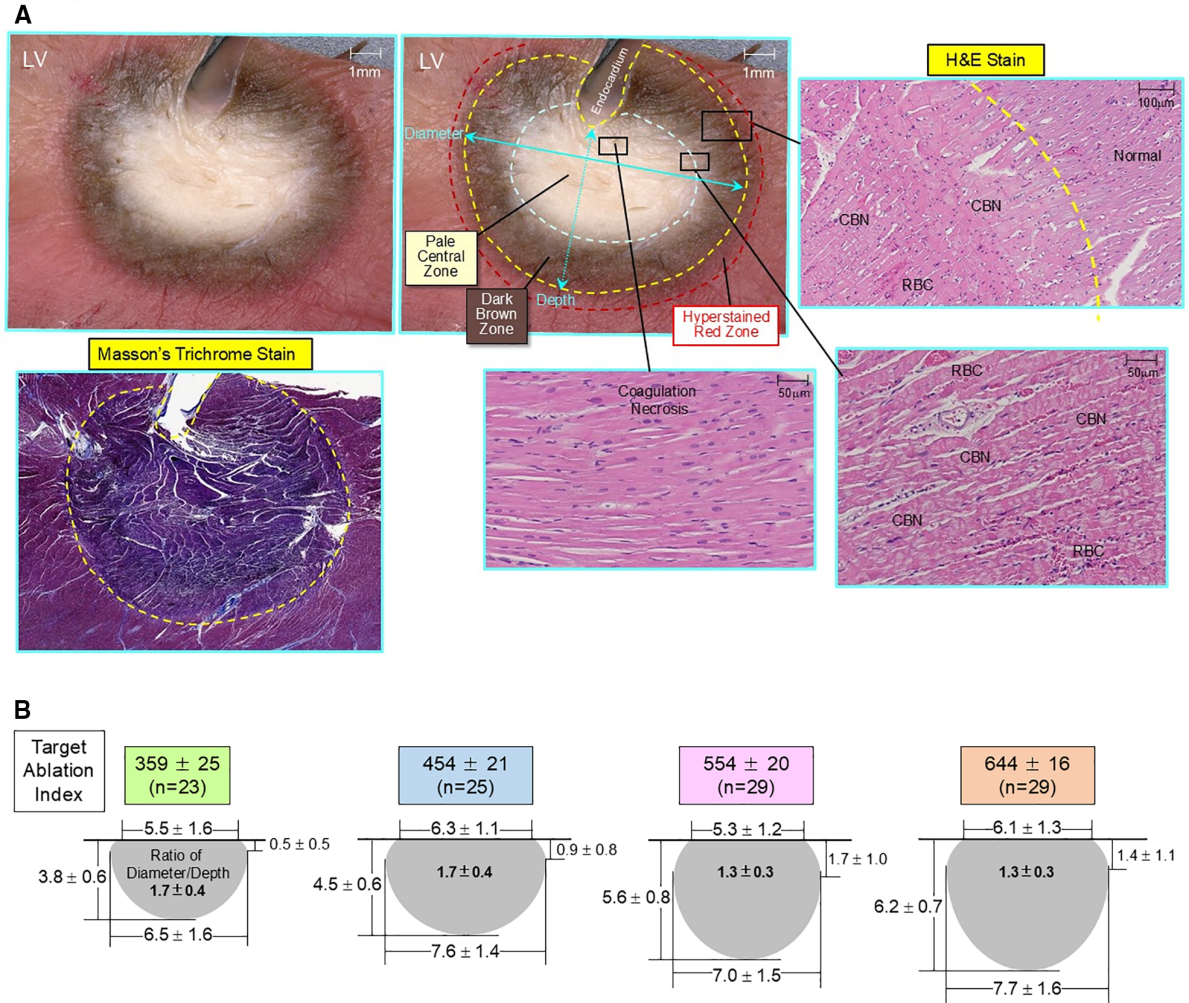
**Histological examination of acute pulsed field ablation (PFA) lesions and measurements of lesion size. A**, A cross-section of the ablation lesion in the left ventricle (LV) with triphenyl tetrazolium chloride (TTC)-staining 2 hours after PFA (12 burst pulse applications with average contact force [CF] of 24 g) with the ablation index value of 551 (predicted lesion depth of 5.51 mm), demonstrating 3 zones: (1) a pale central zone, (2) a dark brown zone, and (3) a hyperstained red zone. Microscopic examination (hematoxylin and eosin [H&E] staining) of these 3 zones demonstrates: (1) contraction band necrosis (CBN) with minimal coagulation necrosis located just below the endocardium; (2) CBN with hemorrhage (red blood cell [RBC]) and increased interstitial space (edema); and (3) unaffected normal myocardium, respectively. Irreversible lesion depth and diameter are measured by combining the pale central zone (dashed gray line) and the dark brown zone (dashed yellow line) with a maximum depth of 5.45 mm and a maximum diameter of 9.45 mm, while the hyperstained red zone (dashed red line) is considered as a reversible zone. Masson trichrome staining (left lower panel) of the same lesion shows the margin between the dark blue zone (contraction band necrosis) and the purple zone (unaffected normal myocardium), which corresponds to the margin between the dark brown zone and the hyperstained red zone with TTC-staining (dashed yellow line), identifying an irreversible lesion boundary. **B**, Lesion size (maximum depth, maximum diameter, surface diameter, depth at the maximum diameter, and the ratio of diameter/depth) is shown, divided by target PF Ablation Index values of 359±25, 454±21, 554±20, and 644±16, respectively. See the text for details.

Two hours after completion of PFA, 250 mL of 1% triphenyl tetrazolium chloride (TTC) was intravenously administered. TTC stains red the mitochondrial enzyme succinate dehydrogenase of living cells, distinguishing viable (red) and nonviable (pale) tissue (Figure 4).^18,19^ The hearts were excised and fixed in 10% formalin. The ventricles were sectioned in 2 to 3 mm slices perpendicular to the intraventricular groove (from apex to base). All ventricular sections were scanned using a digital microscope (VHX-5000, Keyence, Elmwood Park, NJ), where each of the ablation lesions was identified using the 3D electroanatomical maps. Maximum depth, maximum diameter, surface diameter, and depth at the maximum diameter were measured for each lesion by 2 investigators blinded to the ablation parameters (Figure 4). Histological examination of the ablation lesions was performed using hematoxylin and eosin staining and Masson’s trichrome staining (Figure 4).^20,21^

### Statistical Analysis

Statistical analysis was performed using Stat View (ver.5.0) and JMP (version 16.2.0, SAS Institute Inc, Cary, NC). Continuous variables are presented as mean±SD for normally distributed variables. Median with range and interquartile range are also shown for non-normally distributed variables. Categorical variables are described as absolute values and percentages. The comparison between categorical variables was performed with the χ^2^ test or the Fisher exact test, as indicated.

Bipolar and unipolar ventricular potential amplitude (peak-to-peak), unipolar ventricular potential mean negative *dV/dt* of the downstroke, and unipolar ST-segment amplitude (injury current) were compared before and after PFA using paired *t* test or Wilcoxon signed-rank test, as appropriate. The relationship between predicted lesion depth by the PF Ablation Index and actual lesion depth was assessed. The relationships between lesion size (maximum depth and maximum diameter) versus the ratio of post/pre-PFA of bipolar and unipolar ventricular electrogram amplitude, mean negative d*V*/d*t*, and ST-segment amplitude were assessed by simple regression analysis. The relationships between lesion size, the impedance decrease, and the maximum electrode temperature were also analyzed using simple regression analysis. The significance of correlation was determined based on the Pearson correlation coefficient (R-value) and the coefficient of determination (R^2^ value). A probability value (*P*) of <0.05 was considered statistically significant.

## Results

In 6 swine, biphasic monopolar PFA applications were delivered between the ablation electrode and a skin patch at 123 separate ventricular sites (65 LV and 58 RV sites). PFA applications were delivered with electrode-myocardium contact at 111 of the 123 sites at low CF (6–14 g, 11.3±2.4 g, n=25), moderate CF (16–30 g, 23.4±4.6 g, n=43), high CF (31–45 g, 37.1±4.0 g, n=28), or very high CF (48–68 g, 54.9±6.8 g, n=15; Figure 1). In the remaining 12 sites (6 LV and 6 RV sites), PFA applications were delivered without electrode-myocardium contact ≈1 to 2 mm away from the endocardium (confirmed by ICE and CF recordings) with 24, the maximum number of PFA burst pulses (Figures 1 and 2).

In 111 ablation sites with electrode-myocardium contact, targeted PF Ablation Index values were around 350 (mean, 356±29) in 26 RV sites and 1 LV site, 450 (454±21) in 25 RV sites, 550 (554±20) in 29 LV sites, and 650 (644±16) in 30 LV sites, respectively. The number of PFA burst pulses delivered to reach the target PF ablation index values was 11.6±5.5 for the ablation index of around 350, 13.4±4.0 for the ablation index of around 450, 14.7±4.6 for the ablation index of around 550, and 16.9±4.5 for the Ablation Index of around 650, respectively.

ICE monitoring showed no increase in microbubble formation during PFA applications (Figure 2C, right lower panel). No muscle contraction was observed during PFA. Neither steam pop nor impedance rise (defined as >10 Ohm increase from the minimal impedance value) was observed during PFA applications. For the 123 PFA, only 1 episode of ventricular fibrillation occurred after a PFA application in the LV (12 burst pulses at an average CF of 26 g and the calculated ablation index value of 542). After PFA, no thrombus was present on the ablation electrode or on the endocardium at any ablation site by ICE or postmortem examination.

### PFA Lesion Size Measurements and Histological Examination

TTC-staining clearly identified all of the 111 PFA lesions with electrode-myocardium contact (CF: mean 28.4±14.5 g, range of 4–68 g) macroscopically. In contrast, there were no detectable lesions at any of the 12 PFA sites without electrode-myocardium contact, ≈1 to 2 mm away from the endocardium, despite applications with full 24 PFA burst pulses.

The ablation lesions were well demarcated with TTC-staining, demonstrating 3 distinct zones (1) a pale central zone, (2) a dark brown zone, and (3) a hyperstained red zone (Figure 4A). Microscopic histological examination using hematoxylin and eosin staining of these 3 zones demonstrated (1) contraction band necrosis with minimal focal coagulation necrosis (located in the center of the lesion, just below the endocardium) in the pale central zone with TTC staining; (2) contraction band necrosis with nuclear pyknosis and hemorrhage (red blood cell) and increased interstitial space (edema) in the dark brown zone with TTC staining; and (3) no apparent histological changes (unaffected normal myocardium) in the hyperstained red zone with TTC staining, respectively (Figure 4A).^16,17^ Microscopic histological examination with Masson’s trichrome staining of the PFA lesions demonstrated a dark blue zone surrounded by a purple zone. The dark blue zone corresponded to the pale central zone and the dark brown zone on TTC staining (Figure 4A).^16,17^

Lesion size (maximum depth, maximum diameter, surface diameter, and depth at the maximum diameter) was measured by combining the pale central zone and the dark brown zone on TTC staining as irreversible lesion boundaries (Figure 4A and 4B). One hundred and 6 of the 111 lesions (95%) produced with electrode-tissue contact were nontransmural, and the remaining 5 lesions (5%) were transmural. Lesion size measurements were excluded for these 5 transmural lesions because the values would be artificially low (4 lesions in the RV and 1 lesion in LV).

For the 106 nontransmural lesions, lesion size (maximum depth, maximum diameter, surface diameter, and depth at the maximum diameter) is shown in Figure 4B, divided by target PF Ablation Index values. By increasing target PF Ablation Index values, the maximum lesion depth significantly increased: 3.8±0.6 mm for the Ablation Index of 359±25, 4.5±0.6 mm for the Ablation Index of 454±21, 5.6±0.8 mm for the Ablation Index of 554±20, and 6.2±0.7 mm for the Ablation Index of 644±16, *P*<0.001, respectively. The maximum diameter was significantly smaller for the Ablation Index of 359±25 compared with lesions produced by the Ablation Index of 644±16 (6.5±1.6 mm versus 7.7±1.6 mm; *P*<0.05). There was no significant difference in the surface diameter among the 4 groups. The depth at maximum diameter was significantly smaller with the Ablation Index of 359±25, compared with lesions produced by the Ablation Index of 554±20 and 644±16 (0.5±0.5 mm versus 1.7±1.0 mm and 1.4±1.1 mm, respectively; *P*<0.01). The ratio of maximum diameter to maximum depth (ratio of diameter/depth) was significantly greater with the Ablation Index of 359±25 and 454±21, compared with lesions produced by the Ablation Index of 554±20 and 644±16 (1.7±0.4 and 1.7±0.4 versus 1.3±0.3 and 1.3±0.4, respectively; *P*<0.001; Figure 4B).

### Relationship Between PF Ablation Index Predicted Lesion Depth and Actual Lesion Depth

**Figure 5.**
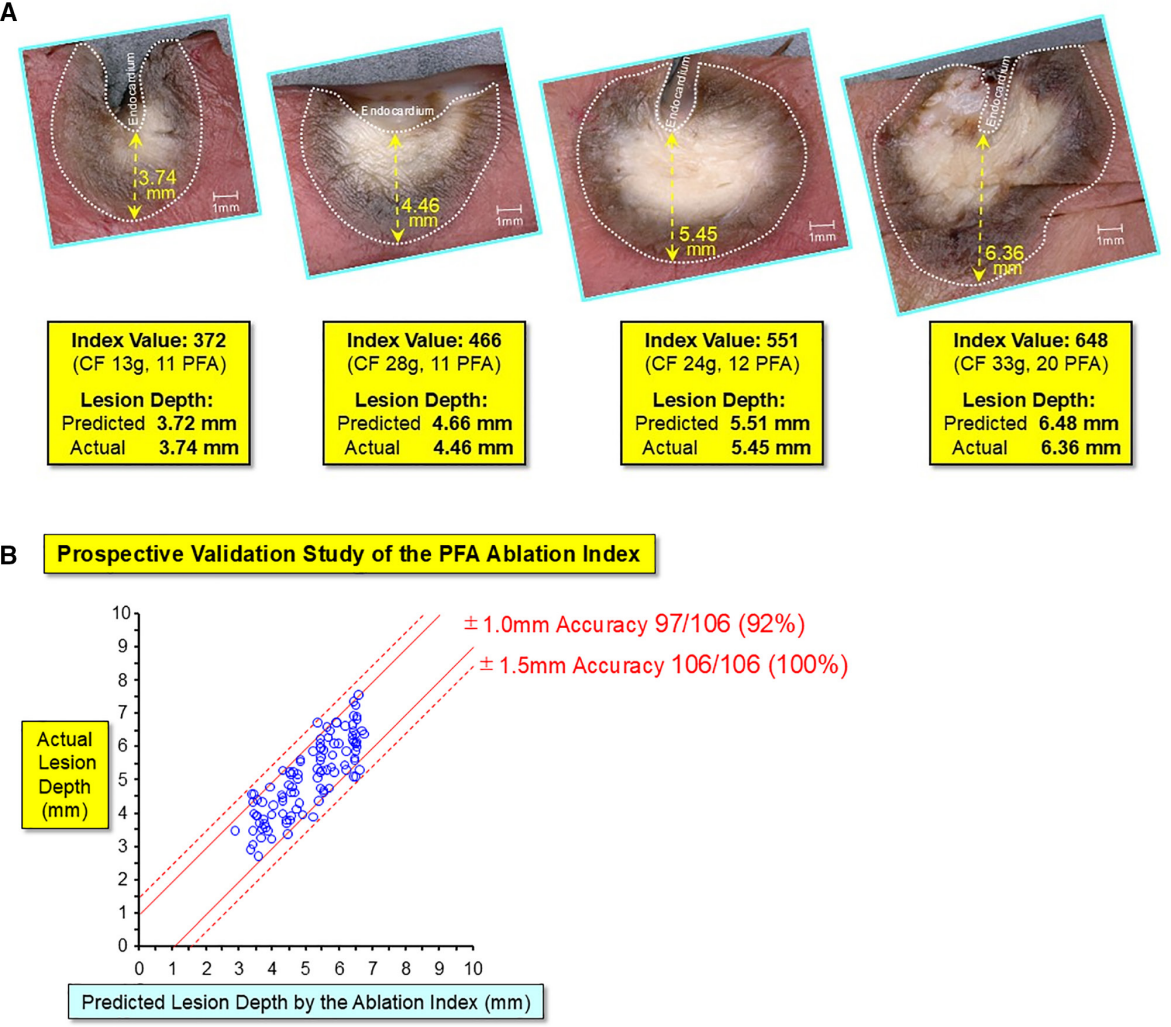
**Relationship between pulsed field (PF) Ablation Index predicted lesion depth and actual lesion depth. A**, Cross-section of the ablation lesions to compare the PF Ablation Index predicted lesion depth and actual lesion depth. The lesion depth created with the target PF Ablation Index values of 372 (predicted lesion depth of 3.72 mm), 466 (4.66 mm), 551 (5.51 mm), and 648 (6.48 mm) resulted in actual lesion depth of 3.74 mm, 4.46 mm, 5.45 mm, and 6.36 mm, respectively, showing that the predicted lesion depths are very close to the actual lesion depths. **B**, The relationship between PF Ablation Index predicted lesion depth and actual lesion depth, indicating the accurate prediction of lesion, with ±1.0 mm prediction accuracy in 97/106 (92%) lesions and ±1.5 mm prediction accuracy in all 106 (100%) lesions. See the text for details. CF indicates contact force.

Predicted lesion depth by the PF Ablation Index correlated well with actual lesion depth, with ±1.0 mm accuracy in 97/106 (92%) lesions and ±1.5 mm accuracy in all 106 (100%) lesions, with the range of actual lesion depth of 2.7 to 7.6 mm (median, 5.2 mm) and ablation index values of 271 to 675 (median, 534; Figure 5A and 5B; Table S1).

### Relationship Between Changes of Intracardiac Electrogram Parameters and Lesion Size

**Figure 6.**
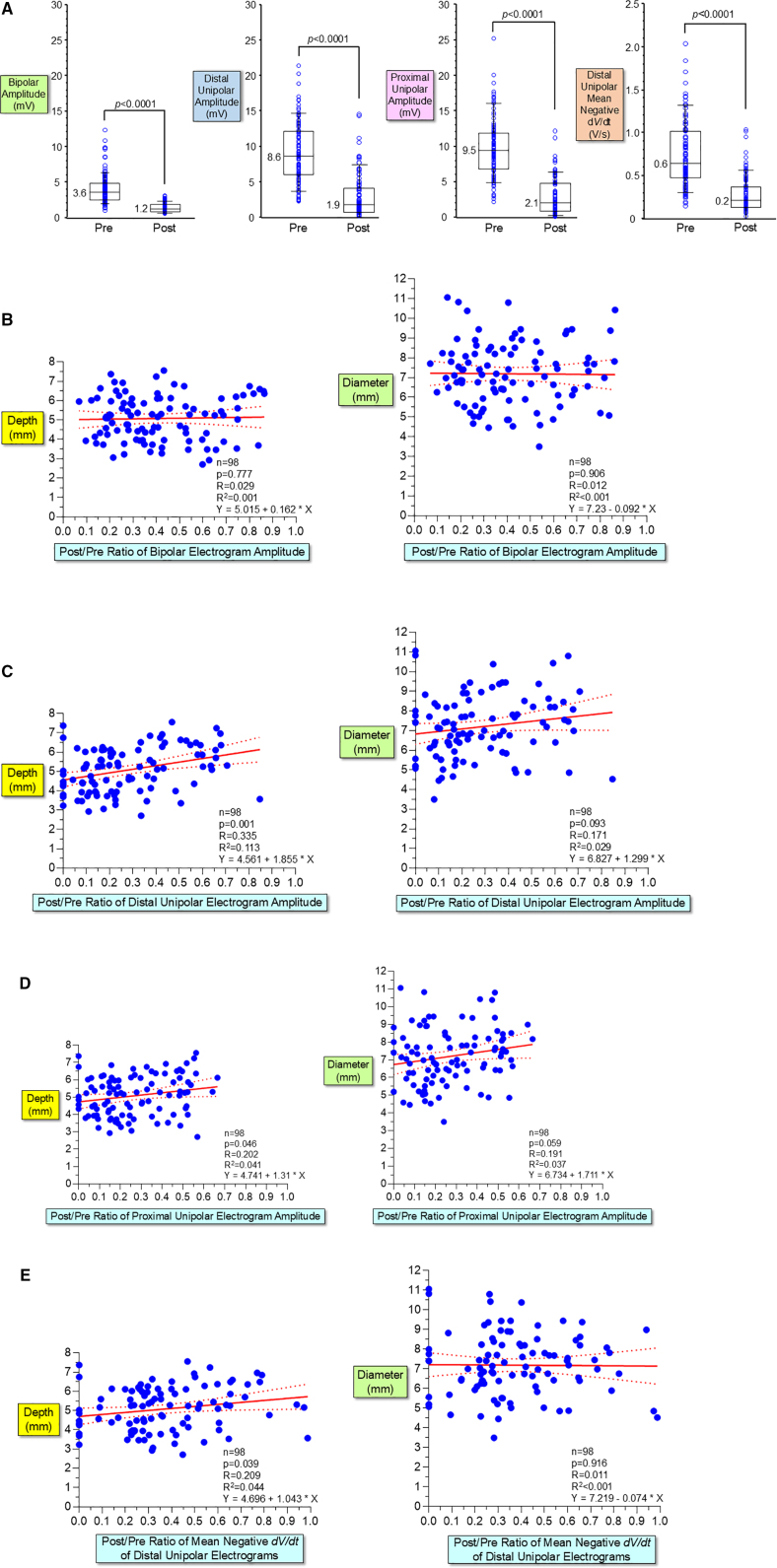
**Relationship between attenuation of intracardiac electrograms after pulsed field ablation (PFA) and lesion size. A**, The peak-to-peak amplitude of bipolar electrograms and unipolar electrograms (recorded from the distal and second electrodes) and the mean negative *dV/dt* of the downstroke of the distal unipolar electrograms significantly decreased after PFA (*P*<0.0001, respectively). **B**, No significant relationship existed between the ratio of post/pre-PFA of the bipolar electrogram amplitude and lesion depth and diameter (*R*=0.029, *P*=0.777 and *R*=0.012, *P*=0.906, respectively). The 95% confidence bounds are shown with dotted lines. **C**, A poor relationship between the ratio of post/pre-PFA of the distal unipolar electrogram amplitude and lesion depth (*R*=0.335), and no significant relationship between the ratio of post/pre-PFA of the distal unipolar electrogram amplitude and lesion diameter (*R*=0.171). **D**, A poor relationship existed between the ratio of post/pre-PFA of the proximal unipolar electrogram amplitude and lesion depth, and no significant relationship with lesion diameter (*R*=0.202, *P*=0.046 and *R*=0.191, *P*=0.059, respectively). **E**, A poor relationship between the ratio of post/pre-PFA of mean negative *dV/dt* of the downstroke in the distal unipolar electrogram and lesion depth (*R*=0.209, *P*=0.039), and no significant relationship with lesion diameter (*R*=0.011, *P*=0.916).

The intracardiac electrograms were obtained before and after PFA applications in 98 of the 106 (93%) nontransmural lesion sites. Compared with before the onset of PFA application, bipolar ventricular potential amplitude (peak-to-peak), unipolar ventricular potential amplitude (peak-to-peak) from the distal and second electrodes, and mean negative *dV/dt* (amplitude/duration of the downstroke slope) from the distal unipolar electrogram significantly decreased after PFA application (*P*<0.0001; Figures 3 and 6A).

There was no significant relationship between the ratio of post/pre-PFA of the bipolar electrogram amplitude and lesion depth or diameter (*R*=0.029, *P*=0.777 and *R*=0.012, *P*=0.906, respectively; Figure 6B). There was a weak relationship between the ratio of post/pre-PFA of the distal unipolar electrogram amplitude and lesion depth (*R*=0.335), and there was no significant relationship between the ratio of post/pre-PFA of the distal unipolar electrogram amplitude and lesion diameter (*R*=0.171; Figure 6C). A poor relationship existed between the ratio of post/pre-PFA of the proximal unipolar electrogram amplitude and lesion depth, and no significant relationship with lesion diameter (*R*=0.202, *P*=0.046 and *R*=0.191, *P*=0.059, respectively; Figure 6D). There was a weak relationship between the ratio of post/pre-PFA of mean *dV/dt* of the distal unipolar electrogram and lesion depth (*R*=0.209, *P*=0.039), but no significant relationship with lesion diameter (*R*=0.011, *P*=0.916; Figure 6E).

Compared with the LV ablation (median lesion depth of 6.0 mm), the ratio of post/pre-PFA of the distal and proximal unipolar electrogram amplitude was significantly smaller in the RV ablation (median lesion depth of 4.1 mm). The ratio of post/pre-PFA of mean *dV/dt* of the distal unipolar electrogram was also significantly smaller in the RV (Figure S1).

**Figure 7.**
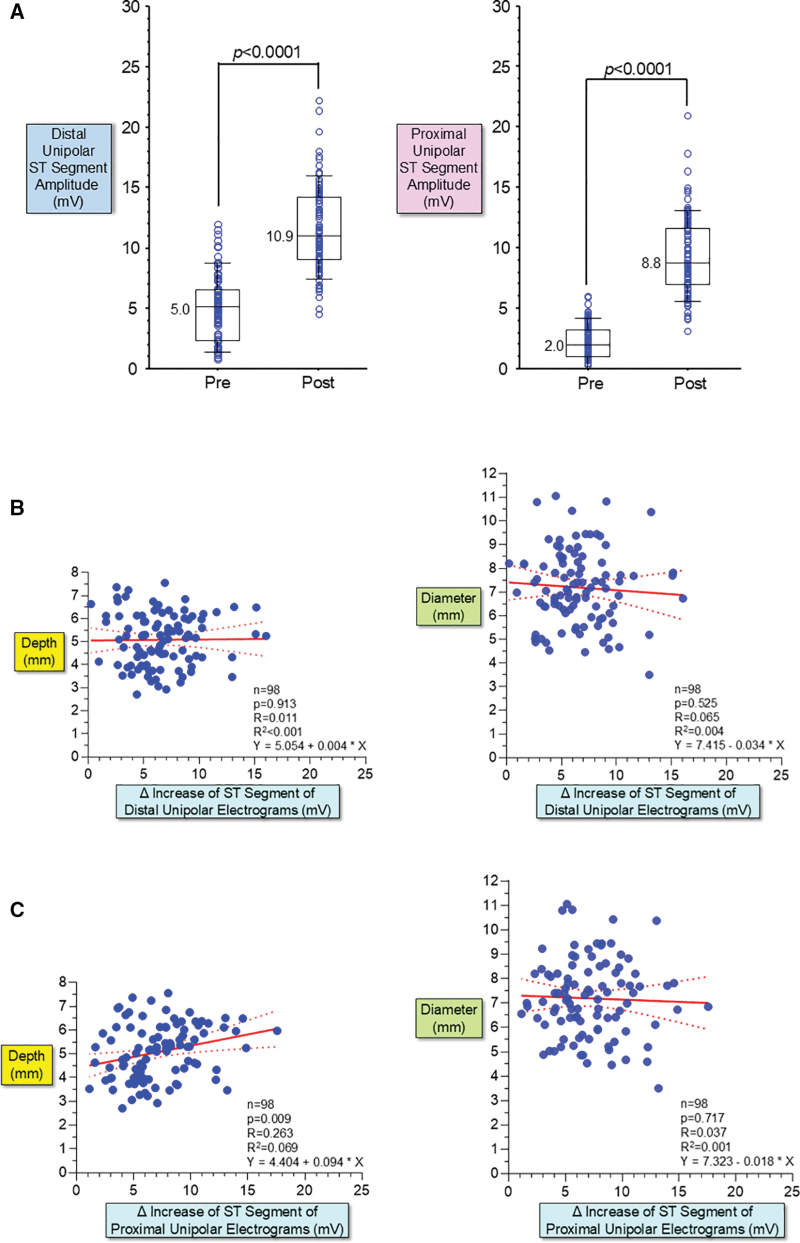
**Relationship between unipolar ST-segment–elevation and lesion size. A**, Unipolar ST-segment amplitude (injury current) recorded from the distal and second electrodes significantly increased after pulsed field ablation (PFA) applications (*P*<0.0001, respectively). **B**, No significant relationship existed between the delta increase of ST-segment amplitude of the distal unipolar electrogram and lesion depth and diameter. The 95% confidence bounds are shown with dotted lines. **C**, A weak correlation existed between the delta increase of ST-segment amplitude of the proximal unipolar electrogram and lesion depth (*R*=0.263, *P*=0.009), but no significant relationship with lesion diameter (*R*=0.037, *P*=0.717).

Unipolar ST-segment amplitude (injury current) from the distal and second electrodes significantly increased after PFA applications (*P*<0.0001, respectively, Figures 3 and 7A). No significant relationship existed between the delta increase of ST-segment amplitude of the distal unipolar electrogram and lesion depth and diameter (Figure 7B). There was a weak correlation between the delta increase of ST-segment amplitude of the proximal unipolar electrogram and lesion depth (*R*=0.263, *P*=0.009), but no significant relationship with lesion diameter (*R*=0.037, *P*=0.717; Figure 7C).

### Relationship Between Impedance Decrease, Electrode Temperature, and Lesion Size

**Figure 8.**
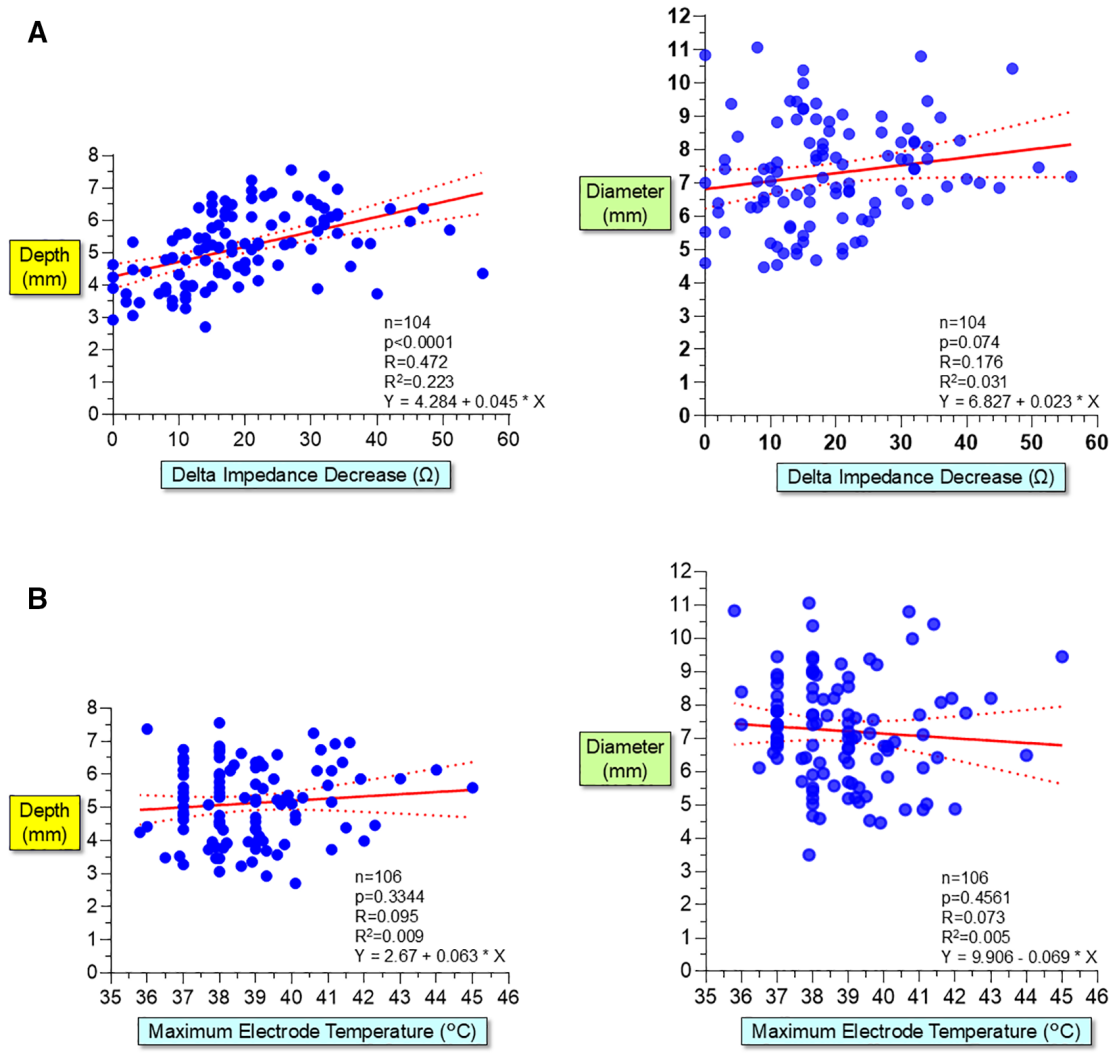
**Relationship between impedance decrease, electrode temperature and lesion size. A**, There was a weak correlation between the magnitude of impedance decrease and lesion depth (*R*=0.472, *P*<0.0001), but no significant relationship with lesion diameter (*R*=0.176, *P*=0.074). **B**, No significant relationship existed between the maximum electrode temperature and lesion depth and diameter (*R*=0.095 and *R*=0.073, respectively).

An impedance (measured between the ablation electrode and the skin patch) was recorded during PFA in 121 of the 123 (96%) PFA applications. The impedance decrease during PFA was 17.5±12.7 Ohms. There was a weak correlation between the magnitude of impedance decrease and lesion depth (*R*=0.472), and there was no significant relationship between the decrease in impedance and lesion diameter (*R*=0.176; Figure 8A).

Electrode temperature was recorded during PFA in all 123 PFA applications. The maximum electrode temperature during PFA with saline irrigation of 4 mL/min was 38.7±1.8 °C. No significant relationship existed between the maximum electrode temperature and lesion depth and diameter (*R*=0.095 and *R*=0.073, respectively; Figure 8B).

## Discussion

Using the biphasic monopolar focal PFA system in a closed-chest beating heart swine model, the present study demonstrated that the PF Ablation Index predicted lesion depth correlated well with ±1.0 mm accuracy in 97/106 (92%) lesions and ±1.5 mm accuracy in all 106 lesions. No detectable lesions were produced without electrode-tissue contact. There was no or only a poor relationship between changes in the intracardiac electrogram after PFA (attenuation of bipolar and unipolar electrogram amplitude or means negative *dV/dt*, or ST-segment–elevation) and lesion size, and no or only a poor correlation between impedance decrease or maximum electrode temperature and lesion size.

### Characterization of Acute PFA Lesions

Similar to the previous study using TTC staining,^15–17^ the acute PFA lesions 2 hours after PFA applications demonstrated well-demarcated lesion boundaries with 3 distinct zones: (1) a pale central zone, (2) a dark brown zone, and (3) a hyperstained red zone (Figure 4A). Microscopic histological examination with hematoxylin and eosin staining of the pale central zone and dark brown zone showed contraction band necrosis with minimal coagulation necrosis, and contraction band necrosis with hemorrhage (red blood cell infiltration), respectively. These 2 zones indicate the irreversible lesion boundary.^15–17^ Histological examination of the TTC-hyperstained red zone at the most outer boundary shows unaffected normal myocardium with preserved mitochondrial activity (evident with positive TTC staining), indicating a reversible zone (presumably areas of transient conduction block).^16–19^

### Prospective Validation of a Novel PF Ablation Index to Predict Lesion Depth

To our knowledge, this is the first study to prospectively validate the accuracy of the PF Ablation Index to predict lesion depth in a beating heart model using this system. PF Ablation Index predicted lesion depth (index values: range of 271–675; median, 534; comprised of CF: range of 6–68 g; median 27 g; and number of burst pulses per application: range of 6–24, median 13) correlated well with actual lesion depth with ±1.0 mm accuracy in 97/106 (92%) lesions and ±1.5 mm accuracy in all 106 (100%) lesions, covering the wide range of lesion depth of 2.7 to 7.6 mm (Figure 5A and 5B; Table S1).

In clinical studies using the radiofrequency ablation index for PV isolation, target index values of 350 to 400 (predicted lesion depth of 3.5–4.0 mm) for the posterior/inferior left atrial wall and 550 to 600 (predicted lesion depth of 5.5–6.0 mm) for the anterior/superior wall have been shown to achieve complete ipsilateral PV isolation at a high incidence with the first encirclement.^5,6^ The present study demonstrates that the PF Ablation Index can accurately predict a similar range of lesion depth (3–7 mm), suggesting its potential to facilitate PV isolation and the formation of transmural, continuous atrial lesions.

Consistent with previous studies,^16,17^ there were no detectable lesions in any of the 12 PFA sites without electrode-myocardium contact, ≈1 to 2 mm away from the endocardium, even with the maximum 24 PFA burst pulse applications, suggesting the necessity of electrode-tissue contact for irreversible lesion formation.

### Relationship Between Changes of Intracardiac Electrogram Parameters and Lesion Size

This is also the first study to systematically examine changes in intracardiac electrograms recorded from the ablation catheter induced by PFA. Although the amplitude of bipolar and unipolar intracardiac electrograms and the mean negative *dV/dt* (slope) of the downstroke of unipolar electrograms significantly decreased after PFA, there was no or poor relationship between the degree of attenuation of these electrograms and lesion size (Figures 3 and 6). Similarly, the ST-segment amplitude (injury current) significantly increased after PFA, but no or poor relationship existed between the increase of ST-segment amplitude and lesion size (Figure 7). These changes in intracardiac electrograms are identified in both distal and proximal unipolar electrograms in all PFA sites, suggesting that PFA leads to a relatively large area of attenuation in intracardiac electrograms regardless of the size of the irreversible lesion. Changes in intracardiac electrograms do not predict PFA lesion size, further highlighting the importance of the PF Ablation Index for real-time prediction of irreversible lesion size.

As with previous studies, the present study suggests that the outermost layer of PFA lesions represents a reversible zone, indicated by the TTC-hyperstained zone.^16,17,19^ This reversible zone is thought to form a transient conduction block zone, and its extent likely exceeds the boundaries of the TTC-hyperstained zone.^8,9,15–17,19,22,23^ Several in vitro experimental studies have shown that reversible electroporation on myocytes induces transient depression of excitation, resulting in a reduction of resting membrane potential, action potential amplitude, and upstroke (*dV/dt*) in a large area.^22–29^

To prevent transmural lesion formation, PFA targeting a lesion depth of 3.5 to 4.5 mm was performed in the RV, while PFA targeting a lesion depth of 5.5 to 6.5 mm was performed in the LV. However, the degree of intracardiac electrogram attenuation following PFA was significantly greater in the RV (Figure S1), suggesting that the remaining intracardiac electrograms after PFA primarily result from far-field potentials originating from outside the lesion. Therefore, even though deeper lesions are created in the LV, the greater wall thickness of the LV likely results in a larger residual far-field potential. The influence of far-field potentials after PFA would be reduced in the thinner atrial wall near the PVs, resulting in greater attenuation of atrial electrograms or complete elimination of PV potentials following PFA.^13,14^

Changes in epicardial ventricular electrograms during epicardial PFA using a focal ablation system were examined with multipolar plaque electrodes in a swine model.^30^ Significant attenuations in the unipolar electrogram (reduction of unipolar electrogram amplitude and maximum negative *dV/dt*, and ST-segment–elevation) were observed after PFA applications. Although epicardial potentials partially recover over time following PFA applications, no significant differences in unipolar electrogram changes were identified at the boundary between irreversible and reversible lesions at 1 minute, 5 minutes, and 60 minutes after PFA delivery, indicating the limitations of estimating the irreversible PFA lesion size based on electrogram changes during PFA procedures.^30^

### Relationship Between Impedance Decrease, Electrode Temperature, and Lesion Size

There was only a weak correlation between the magnitude of impedance decrease and lesion depth, and no significant correlation with lesion diameter (Figure 8A). The mechanism of impedance decrease by PFA is thought to involve the pore formation in the highly insulating lipid bilayer of the cell membrane (increased membrane permeability) and enhanced electrical conductivity of the myocardium due to minimal tissue heating. However, since impedance decrease is observed early after the initiation of PFA application and plateaus relatively quickly, its correlation with lesion size is considered to be limited.^16,17^

The increase in myocardial temperature caused by PFA is limited, resulting in minimal elevation of electrode temperature (maximum temperature of 38.7±1.8 °C). Consequently, no significant relationship is observed between the maximum electrode temperature and lesion depth and diameter (Figure 8B). Histological examination also indicates that thermal injury, characterized by coagulation necrosis, is confined to a small area directly beneath the ablation electrode. Most of the ablation lesion demonstrates contraction band necrosis, which is considered a nonthermal injury.^12,15–17^

### Clinical Implications

The creation of irreversible PFA lesions is desired to ensure lesion durability. However, recent clinical studies have demonstrated frequent PV reconnections in the follow-up PV mapping studies despite acute complete PV isolation at the initial PFA procedure.^31–35^

The present study suggests that the reduction or elimination of intracardiac electrograms induced by PFA encompasses not only the formation of irreversible lesions at the electrode-myocardial contact site but also a relatively large surrounding area of reversible lesions (transient conduction block zone).^16,17,19,23^ Therefore, it is challenging to estimate the extent of irreversible lesions based on the reduction or elimination of intracardiac electrograms after PFA. It is feasible to accurately predict irreversible lesion depth in real-time using the new PF Ablation Index incorporating CF and the number of burst pulses. Lesion diameter may also be estimated based on the ratio of diameter to depth: the lesion diameter would be ≈1.7× the depth for Ablation Index values of 350 to 450, and the diameter would be ≈1.3× the depth for Index values in the range of 550 to 650 (Figure 4B). Therefore, PFA guided by the novel Ablation Index may facilitate the creation of continuous transmural lesions without excessive, unnecessary PFA applications.

PFA without electrode-myocardial contact would fail to create effective irreversible lesions and may increase the risk of hemolysis and microbubble formation.^36–39^ To reduce the risk of complications (including kidney injury and stroke), unnecessary and frequent PFA applications should be minimized, especially when the electrode is not in contact.^36,38,39^

### Study Limitations

One limitation of this study is that histological examination was performed acutely, 2 hours after PFA. In our other study, we investigated the changes in reversible and irreversible ventricular lesion boundaries at <30 minutes, 1 hour, 2 hours, 4 hours, and 14 days after PFA. Using TTC staining and histological examination, we found that the size of the irreversible lesions remained similar at 2 hours, 4 hours, and 14 days after PFA.^40^ At 2 and 4 hours post-PFA, the irreversible lesion boundaries exhibited contraction band necrosis, while at 14 days post-PFA, these lesions had transitioned to fibrosis with a similar size. Based on these findings, we assessed irreversible lesion size in 2-hour post-PFA ablation lesions using TTC staining and histological examination in the present study.^15–17,40,41^

Intracardiac electrogram changes produced by PFA were examined immediately after PFA applications, and changes in electrograms over time were not investigated.

Another limitation of the present study is that only a biphasic, monopolar focal ablation system was tested. Relationships between the parameters tested and lesion size may differ using different PFA systems. We performed a preclinical study in a swine beating heart model using a large footprint multi-electrode ablation catheter (12 electrodes, 12 mm in diameter) with bipolar PFA applications, resulting in greater lesion depth with increasing CF and PFA doses.^41^

When PFA is performed on normal myocardium and infarcted scar tissue, lesion size is expected to differ even with the same CF and number of pulses. Therefore, a new PF ablation index for scar tissue may be required.

PFA was performed in the LV and RV rather than the atrium to allow accurate measurement of lesion depth in nontransmural ventricular lesions. In in vivo beating heart studies, the expected measurement error for lesion size is ≈±0.5 mm. This error may be considered one of the causes of variability between predicted and actual lesion size.

Finally, clinical studies are required to determine the efficacy and safety of the PFA system using the novel Ablation Index, including the potential reduction of the risk of complications.

### Conclusions

In this prospective validation study using the biphasic monopolar focal PFA system in a closed-chest beating heart swine model, a novel PF Ablation Index (incorporating CF and the number of PFA pulses) provided real-time prediction of PFA lesion depth, with ±1.0 mm accuracy in 97/106 (92%) lesions and ±1.5 mm accuracy in all 106 lesions. No detectable lesions were produced without electrode-tissue contact. There was no or only a poor relationship between changes in the intracardiac electrogram after PFA (attenuation of bipolar and unipolar electrogram amplitude and mean negative *dV/dt,* or ST-segment–elevation) and lesion size, and no or only a poor correlation between impedance decrease or maximum electrode temperature and lesion size.

## ARTICLE INFORMATION

### Sources of Funding

This study was supported, in part, by a grant from Johnson & Johnson MedTech Inc.

### Disclosures

Dr Nakagawa is a consultant for Johnson & Johnson MedTech Inc, Abbott Inc, CardioFocus Inc, Stereotaxis Inc, Japan Lifeline, Ltd, Synaptic Medical Inc, Philips Japan, Ltd, and Fukuda Denshi, Ltd. Dr Jackman is a consultant for Johnson & Johnson MedTech Inc. Dr Farshchi-Heydari, J. Maffre, Dr Sharma, P. Lam, Dr Govari, C.T. Beeckler, and A. Altman are employees of Johnson & Johnson MedTech Inc. The other authors report no conflicts.

### Supplemental Material

Table S1

Figure S1
